# Action-research in the development of a change management model to strengthen health interventions with an intercultural approach, Peru

**DOI:** 10.17843/rpmesp.2025.422.14134

**Published:** 2025-06-09

**Authors:** Gualberto Segovia-Meza, Armando Medina-Ibañez, Marco Bartolo-Marchena, Betty Monteza-Facho

**Affiliations:** 1 Ministry of Health, Lima, Peru. Ministry of Health Lima Peru; 2 Federico Villareal National University, Lima, Peru. Federico Villareal National University Federico Villareal National University Lima Peru; 3 National Center for Social and Intercultural Research in Health, National Institute of Health, Lima, Peru. National Center for Social and Intercultural Research in Health National Institute of Health Lima Peru

**Keywords:** Change Management, Interculturality, Health of Indigenous Peoples

## Abstract

We present our experience in developing a change management model to strengthen intercultural health interventions in primary care. This is an action-research project involving health teams that mainly care for indigenous populations. The methodology included problem-based workshops and deductive analysis. The study was conducted in 44 health facilities in six regions, using a specific guide to build the change model. As a result, a change management model was developed, consisting of three main components: a change management guidance model, a change planning model, and change management meetings based on continuing health education. A change management model is presented to strengthen the prevention and control of health priorities with an intercultural approach, with the potential to improve the effectiveness of health interventions.

## INTRODUCTION

An analysis of 17 American countries in 2023 on the challenges facing the health system estimated that 34.4% of the population had unmet health care needs, a situation that mostly affected the poorest quintile. Some of the causal factors in that study were a lack of trust in health personnel, insufficient intercultural approaches, and linguistic barriers ^1^. The COVID-19 pandemic undoubtedly had a negative impact on health programs in American countries. A review of the impact of the pandemic on concurrent infections identified tuberculosis (TB) and COVID-19 as two of the leading causes of death worldwide ^2^^,^^3^.

Before the pandemic, the health system was characterized by its low efficiency. In 2018, PAHO/WHO conducted a comparison of TB incidence rates among indigenous peoples in six American countries with the general population; it showed large differences, with rates among ethnic groups exceeding those of the general population by 2 to 10 times ^4^. This situation was the result of a health system with serious problems in addressing health priorities. It was not until 2024 that PAHO/WHO approved strategies to strengthen essential public health functions with the aim of accelerating the transformation of health systems between 2024 and 2034 ^5^.

Health interculturality is a big challenge in Peru; we are a multicultural and multiethnic country with 47 indigenous languages and rich archaeological heritage. According to the 2017 census, 24.9% of the general population identified themselves as indigenous. This diversity requires us to promote an intercultural approach, with respectful intercultural dialogue and mutual learning ^6^^,^^7^. There are regulations that promote interculturalism in health; however, there are weaknesses in their implementation.

Indigenous peoples are considered a vulnerable population. In 2022, the Ministry of Health evaluated the health management of the tuberculosis control program in the indigenous population for 2017-2022 and identified that 1,461 people were indigenous, 59.3% of whom were Amazonian indigenous and 40.7% Andean indigenous. There was an increase in the number of cases from 2019 to 2022, related to the effects of the COVID-19 pandemic ^8^.

A central element of efficient health systems is primary health care; we need to promote the development of our health systems to improve equity and move toward universal health coverage (PAHO/WHO). The shortcomings of our health system became evident with the emergence of health emergencies, such as monkeypox in 2022 and the dengue outbreak in 2023, when our health system struggled to respond comprehensively. Within a few weeks, most regions of Peru reported confirmed cases of monkeypox, and we reported 381 deaths from dengue ^9^^,^^10^.

An evaluation of primary health care management in Peru (2022), in health facilities that cared mainly for indigenous populations, identified barriers to the prevention and control of the COVID-19 pandemic. These barriers were related to vision, management, and budgeting in care for indigenous and Afro-Peruvian populations; this study recommended applying a change management model with an intercultural approach with the aim of achieving greater access to health services ^11^. In the same year, 2022, PAHO/WHO made recommendations to transform health systems and suggested implementing the Change Management Model based on Kotter’s steps ^12^.

Change management means promoting a new form of organizational development with the aim of improving productivity or providing a higher standard of living. Developing this transformative process involves many agents of change, with the work team leading the plan to be introduced ^13^. According to Kotter and Bridges, this transformative process involves steps that require considerable time, and a mistake at any stage can have devastating effects. Changes must even take place within individuals ^14^^,^^15^. Likewise, innovation strategies are being tested in healthcare, such as the Organizational Innovation Management Model (EMOI), which allows for understanding customer needs, defining innovation, and applying it with an innovation plan ^16^.

Currently, we do not have a clearly defined management model to promote organizational change in the health sector. The contribution of PAHO/WHO in 2022, based on the regional forum to expand equitable access to health services, recommended using change management strategies with proven evidence, adapting Kotter’s eight steps to lead change ^15^. A national experience was carried out in 2021, when a team of researchers from the National Center for Intercultural Health adapted a Change Management Model to incorporate an intercultural approach into tuberculosis intervention in the Ica region ^17^.

Based on the above, this paper aimer to present our experience in developing a change management model to strengthen healthcare interventions with an intercultural approach.

## METHODOLOGICAL APPROACH

This article describes the process of developing a change management model using action-research, which is based on constructivism as its epistemological foundation, allowing for an understanding of knowledge construction through practice, with the aim of strengthening health interventions with an intercultural approach ^18^^-^^21^.

### Population and study location

The study was conducted in selected regions with the highest concentration of indigenous populations. The study population consisted of health teams from primary care centers, who were invited to participate in workshops designed to analyze and propose plans for change in health interventions prioritized by the health teams themselves.

Healthcare centers were selected to participate by intentional sampling; one of the criteria was being a healthcare center that serves a predominantly indigenous Andean and Amazonian population. The approach was an official call from the National Center for Intercultural Health to the Regional Health Directorates (DIRESA), which then called on and prioritized healthcare centers that mainly care for indigenous populations. The sample size was 44 primary healthcare centers in six regions: Ica (2021-2022), La Tinguiña Health Center, Parcona, Guadalupe, Pampa Villacuri Health Post; Health Networks: Chincha, Pisco, Ica, Nasca. In Amazonas (2022), the networks: Chachapoyas, Bagua and Condorcanqui, Utcubamba, IPRESS, which care for the Awajun and Wampis populations. In Ayacucho (2022), the Vinchos micro-network, Morochucos micro-network, Ocros micro-network, mainly caring for the Quechua population. Pasco (2022), with the Oxapampa Network. Junín (2022) with Healthcare Service Providers (IPRESS) in Pangoa, Pichanaki, Satipo. Huánuco (2022) with the IPRESS in Leoncio Prado, Puerto Inca, which care for the Ashaninka and Yanesha communities.

### Data collection

Health interventions or strategies were defined by each healthcare center, while in others they were defined by DIRESA according to regional priorities. Workshops were conducted using the problematization methodology, which begins with observation of the reality of the problem, followed by identification of critical points, theorization of the issue, and proposal of possible solutions. The workshop program lasted two days and allowed researchers to obtain information using participant-observation, which consisted of researchers participating in group discussions with motivating questions to encourage the problematization of issues.

The data were collected from the posters created by the healthcare teams, from the plenary sessions, recordings, records of the change-plan guides, interviews with heads of health facilities, workshop reports, observations on the development of the plans, and from the virtual presentations.

All the collected information was systematized based on the objectives of the study, that is, according to the components of the change-management model under development: a) Change-management guidance model. b) Change-planning model. c) Change management meetings based on Continuing Health Education (CHE).

### Procedure

First stage: The first stage started with a plan for the Ica region (2021), “Development of a Change Management Model to strengthen tuberculosis prevention and control with an intercultural approach.” Workshops with health teams from four healthcare centers and the regional health authority were held. The action-research methodology was applied considering the following steps: 1. First cycle: The current situation of the comprehensive management of the tuberculosis program was analyzed during the first cycle using the problematization pedagogy. The healthcare teams identified their problems and needs with regard to user needs, organizational needs, resource management, service delivery, and interculturality. 2. Second cycle. A plan was developed during the second cycle to strengthen tuberculosis prevention and control with an intercultural approach, using an appropriate guide based on the organizational innovation management model (EMOI). 3. Third cycle. Health teams and facility managers were trained to implement the plan; an adaptation of the Continuing Health Education (CHE) strategy was used to implement the plan. 4. Fourth cycle. The implementation actions and effects were evaluated, and improvements or adjustments were made to the original design of the model under development.

Second stage: An action plan was developed to implement the “Change-Management Model for Health Interventions with an Intercultural Approach” in the regions of Ayacucho, Amazonas, Pasco, Huánuco, and Junín. The activities were carried out according to the action-research design; beginning with a first cycle was followed which identified problems in the organization, management, and delivery of health interventions prioritized by the healthcare teams. The addressed topics were: COVID-19, TB, HIV/AIDS, dengue, maternal health, mental health, and monkeypox. The second, third, and fourth cycles followed, collecting data in each one, analyzing and systematizing it, with the aim of developing the study model.

**Figure 1:**
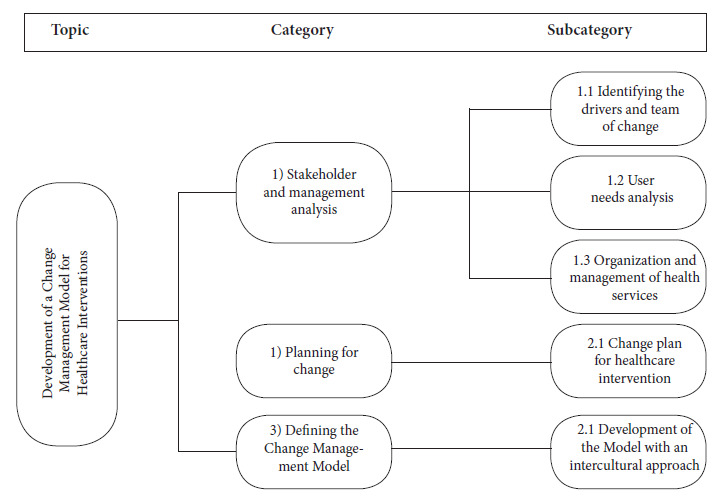
Coding tree for the study: Theme, categories, and subcategories

Third stage: The “Change-Management Model for Health Interventions with an Intercultural Approach” was defined, and all the collected information was used to code qualitative data related to the research problem. What are the elements of the change management model as a tool that will strengthen health interventions with an intercultural approach? Categorization for analysis was carried out through deductive analysis, manually, and interpretation of the data, according to the studied phenomena, defined as three categories: 1. Analysis of stakeholders and management 2. Planning for change 3. Defining the change management model (Figure 1).

### Data analysis

Number of coders. The collected data was coded by the principal researcher, with the assistance of three researchers from CENSI. Coding was performed manually in Microsoft Excel.

Description of the coding tree. The process began by identifying the research topics, then the main categories were created, and finally the subcategories shown in Figure 1.

Verification by participants. Participants presented their analysis of the problem related to the steps of the change-management model appropriate for this study during the regional workshops. They then provided information on the findings of their analysis of user needs, resource management, and difficulties in developing change plans. During the in-person and virtual technical assistance sessions, the healthcare teams presented their complete change plans, and in some cases we verified their implementation.

## REPORTS

**Figure 2:**
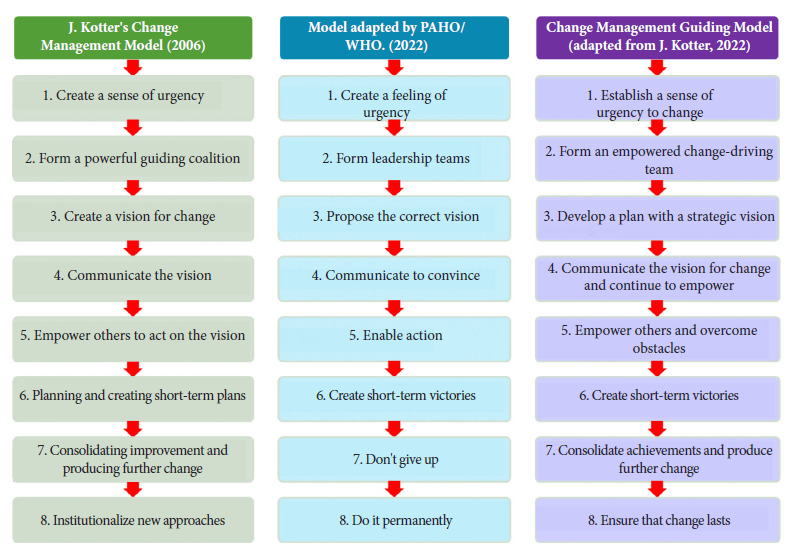
Suitability of the Change-Management Model for intervention as a “Guiding Model for Change Management”.

**Figure 3:**
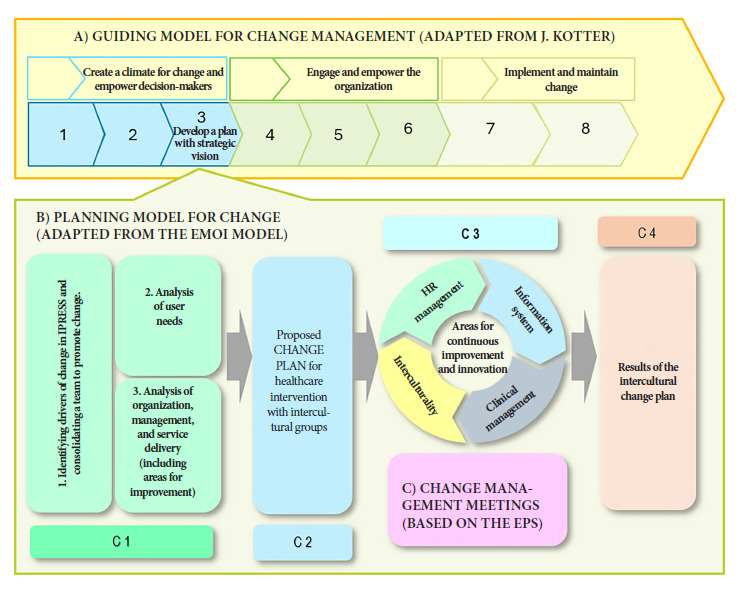
Figure 3. Change Management Model for Health Interventions with an Intercultural Approach.

Change-management model appropriate for the intervention. It is called the “Change management model for health interventions with an intercultural approach” and has three components:

1. Change-management guidance model. After the necessary adjustments based on feedback from the healthcare teams, it was consolidated as a guiding component throughout the change management development process, adapted from Kotter’s model and the model proposed by PAHO/WHO ^(^^1^^,^^14^^)^ (Figure 2).

2. Planning model for change. Adapted from the EMOI, this management model has four components: Component 1 (C1) involves analyzing stakeholders and the management team, analyzing user needs and the organization, and managing and delivering services. Component 2 (C2) involves developing the plan with its organizational and management components, incorporating the four key areas: health human resources management, information systems, clinical management, and interculturality, with a special focus on the correct recording of ethnic codes. Component 3 (C3) represents the space for the development of the plan and its pillars in human resources, information systems, clinical management, and interculturality. Component 4 (C4) evaluates the generated changes (Figure 3).

3) Change management meetings. These were designed based on the Critical Reflection on Continuing Education in Health (CEH), which is an educational and management strategy. These meetings represent the engine of change. The health teams agree on the dates and venues for their management meetings, which are considered a space for dialogue on the implementation of the Plan. In this space, experiences are shared, continuing education in health takes place, and changes are proposed.

### Quotes from participants

Workshops were held to identify problems, collect data, and systematize information during the process of constructing the Change Management Model for Health Interventions with an intercultural approach, in accordance with the established categories and subcategories (Table 1).

**Table 1:** Summary model by categories, subcategories, findings, and lessons learned.

Category	Subcategory / Questions for analysis.	Findings (Quotes)	Lessons learned (Researchers’ summary)
1. Stakeholder and management analysis	1.1 Identifying change drivers and change team.		
	How is a sense of urgency established among managers and technical staff in the DIRESAS/healthcare networks?	*Regional and provincial health authorities lack knowledge of change management. Interculturalism is not valued as important in healthcare services; we need more training*. (Head of HC Guadalupe, Ica)	In DIRESA and the Health Networks, the initial motivation for change is reduced if there are no actions to strengthen leadership, teamwork, and communication improvements. There is more interest among IPRESS management teams in the urgency of implementing changes.
	How is the team leading the TB and dengue prevention and control program in primary care IPRESSs structured?	*“The TB control management team is made up of those responsible for the TB strategy and contractors.”* (Head of HC Pampa Villacuri, Ica) *“In dengue, the team is mainly responsible for metaxenics, health promotion, and environmental health.”* (HC Chincha Baja, Ica)	Most health strategy management teams do not involve other key actors in the patient care process, such as pharmacy managers, laboratory managers, outpatient clinic managers, including community health workers, and community leaders.
	1.2 Analysis of user needs		
	Do you identify the needs of users with TB, dengue, COVID-19 to improve their health? With an ethnic focus? (using the empathy map).	*“The health services do not apply strategies to identify the needs of users with TB, dengue, or COVID-19.”* (Ayacucho Health Network official) *Applying the empathy map allows us to identify problems in access to health services if we do not have a user survey."* (HC speaker, Amazonas)	The empathy map provides a better understanding of the needs of patients with TB, COVID-19, dengue, HIV/AIDS, and allows us to identify our weaknesses in responding to needs with greater ethnic relevance.
	What are the needs of family members of TB patients, with a focus on ethnicity? (The empathy map was used)	*“No information is available on the knowledge and perceptions of the TB patient’s family or their ethnic identity.”* (Licensed PCT at the Parcona Health Center, Ica)	It is recognized that families fear contagion, have seen their household income decline, and are considering returning to their place of origin, “their village.” Health personnel report that they lack skills in family intervention and intercultural approaches.
	What are the needs of internal users (health personnel) for better quality care for users of Health Strategies?	*“We identify the limitations or obstacles faced by health personnel or teams in the health strategy to comply with the relevant regulations. We also identify the relationship with the units and services of this IPRESS, or difficulties in working with families and the community.”* (Presentation by the health team of the Bagua Health Network, Amazonas).	The methodology allows reflection on weaknesses in multidisciplinary work and assessment of interculturality in health to improve access to health services. They recognize weaknesses in interculturality, particularly in the correct recording of ethnic codes.
	1.3 Organization and management of health services		
	How are IPRESS organized and managed to overcome obstacles, delegate responsibilities, and bring about change in the fight against TB and dengue?	*The TB and dengue teams generally work in isolation or in a fragmented manner. The team is hired for TB. The dengue control team consists of the coordinators of metaxenics, environmental health, and health promotion.* (HC La Tinguiña, Ica)	Organize work teams with those involved in the process of providing comprehensive care in cases of TB, dengue, etc. Decision-making and leadership are needed from IPRESS heads and network and DIRESA managers to ensure the sustainability of the teams.
	What is the status of your information system for health strategies for TB, dengue, as well as the recording of ethnic codes for health emergencies such as COVID-19, dengue, and TB?	*“The IPRESS do not have electronic medical records. The TB health strategy has a SIGTB program that does not have the correct ethnic codes. The IPRESS have a heat map for dengue, but it is rarely used for intervention. There are no situation rooms broken down by ethnic groups.”* (Presentation by the HC Pampa Villacuri-Ica team)	Training in the correct recording of ethnic codes has identified people with TB from indigenous communities (in Ica: Pampa Villacuri Healthcare center: Shipibos and Quechuas), and actions have been promoted to ensure greater cultural relevance. Little use of heat maps for dengue. No use of data from districts with low COVID-19 vaccine coverage.
2. Planning for change	2.1 The change plan for healthcare intervention		
	Is it possible to develop a Change Plan to strengthen prevention and control of health priorities, and implement it?	*“We now have a more participatory change plan, but the slow development of the plan and failure to carry out tasks is linked to the low number of management meetings. The technical teams are more committed and interested in implementing improvements such as ethnic code registration, but there are always those who oppose the changes that are being proposed.”* (Presentation by the HC team in Pampa Cangallo-Ayacucho).	DIRESA managers and health networks are not empowered; a specific plan is needed for officials and managers in leadership, governance, and organizational change. The sustainability of the progress made by the technical teams requires support and resources to implement the Change Plan.
	What are the critical points in the development of the Change Plan and change management meetings for the prevention and control of health priorities?	*“Management meetings are not held regularly in the IPRESS to address problems in the prevention and control of health strategies because the schedules of the health teams do not coincide. Other strategies are being considered to better schedule the timetables and other issues.”* (Head of HC Sunampe, Ica).	Meetings are effective when formally convened and always begin with critical reflection on practice. Training is needed on strategies for problem-solving meetings and continuing health education.
3. Defining the Change Management Model	3.1 Development of the Model with an Intercultural Approach		
	Establishing the vision for organizational change.	*“By 2025, the IPRESS of Nieva, Urakusa, and Pagkintsa in the MR Nieva, through their change-driving teams, will promote the detection, prevention, and timely and quality care of HIV/AIDS cases with the participation and monitoring of the community, using an intercultural approach”* (Health Team, Nieva Health Center, Urakusa Health Center, and Pagkintsa Health Center. Amazonas).	The tool (Guide) for developing the Change Plan with the components of the model enables the Change Plan to be developed based on local health priorities, with a view to organizational change and an intercultural approach.

## DISCUSSION

Results show the development of a Change-Management Model for Health Interventions with an intercultural approach, which contains three elements. Its development followed an action research framework, with an initial stage of problem analysis. The second stage involved developing the plan. The third stage involved implementing and evaluating the plan. The fourth stage involved feedback, in which the model was reviewed and the necessary adjustments were made.

Since the beginning of the 21st century, recommendations have been made on how to manage organizational change in the public sector, how to integrate knowledge obtained from multiple theoretical perspectives into practice, and how to develop better models and tools for researching change ^19^. This is no easy task. We reviewed the action research methodology in the field of health. There are some publications on health, but there are undoubtedly more applications in the field of social sciences ^22^^-^^25^.

The purpose of this action research methodology is to provide information to promote social change or transform reality. In other words, it allows the study of social problems to be linked, and knowledge and social change to be achieved simultaneously ^20^. Our study uses the action research methodology to focus on understanding and explaining the process of developing a change management model to strengthen health interventions with an intercultural approach, which should lead to improved effectiveness in the prevention and control of health problems, especially in indigenous populations.

A central theme of the research was the development of studies on Change-Management Models in health. In 2022, a literature review was conducted on the topic of “change management in the context of occupational safety and health management.” In general, there were few studies on the subject, and further studies were recommended ^21^. Other studies have addressed mobile technologies for change management in health organizations ^22^. PAHO/WHO is developing an online course titled “Change Management for Telehealth Services” (2024), which defines the change management model as “the application of a structured process and set of tools to lead the human side of change and achieve desired results.” In this regard, there is little information on change management models suitable for strengthening health interventions with an intercultural approach ^26^^-^^29^.

It is necessary to clarify the importance of a change-management model to enhance the impact of health interventions. Kotter’s work in the field of private organizations with experience in business renewal indicates that “leaders who successfully transform their companies get eight steps of change management right, and they do them in the right order” ^14^. No applications of Kotter’s work were found in the healthcare field. A more concrete approach is provided in the PAHO/WHO document “Change Management in Public Health” (2022), which defines change management as the practice and process of supporting people during change, with an emphasis on helping people modify their behaviors, attitudes, or work processes to achieve the desired goal ^2^^,^^30^^,^^31^. Later, in the document Expanding Equitable Access to Health Services: Recommendations for the Transformation of Health Systems Toward Universal Health Coverage, it proposes an adaptation of Kotter’s eight steps ^1^ (Figure 2) and recommends their application in healthcare.

In this regard, we propose the development of a change management model based on Kotter’s eight steps and the adjustments made by PAHO/WHO. We add two complementary elements: an adaptation to the EMOI ^32^^-^^38^^)^ to develop a change plan; and a strategy to operationalize the plan, “Change Management Meetings,” which represents an educational and management strategy for healthcare services, with a methodology based on Continuing Health Education, developed by María C. Davini (PAHO) as Critical Practice Reflection meetings ^23^.

Reviews on interculturality in health point to the existence of approved regulations on the subject, mainly from the health and culture sectors. The problem lies in putting them into practice ^39^. One of the problems is the complexity of articulating them with scientific medicine. There are problems in understanding concepts such as cultural syndrome, which is not easily understood by conventional medicine practitioners ^40^. During the development phase, some key points were identified where regulations were not being applied ^25^, such as ethnic self-identification and the correct recording of ethnic codes, followed by a failure to analyze information and use information with the ethnic variable to implement improvements. An interesting experience is that of the Pampa Villacuri Health Post in Ica, where the healthcare team became empowered and understood the need to understand the needs of the migrant population. In recent years, they have correctly recorded ethnic codes, analyzed data, and set up a situation room with an intercultural approach, which identified the Quechua and Shipiba populations as the most affected by tuberculosis ^17^. Therefore, in our change-management model, we have identified interculturality as a key objective within the change plan.

Regarding the relationship between the first component of the model, “Change Management Guidance Model,” and the second component, “Change Planning Model,” the first model contains the eight steps outlined by Kotter and allows the organizational change process to be directed. The third step, “Developing a Plan with a Strategic Vision” (Figure 3), includes the development of a change management plan that helps manage the change process and clearly articulates the organizational strategy ^41^^,^^42^. This articulation of two models allowed the development of the change plan for healthcare centers, which considers the scheduling of activities mainly in the initial steps, and in the third step, the development of all the components of the “Planning Model for Change” is included. According to the Center for Quality and Change Management at the Polytechnic University of Valencia, the EMOI model is defined as a methodology that enables the effective deployment of innovation at the strategic, tactical, and operational levels, and that allows for understanding innovation demands, defining innovation, deploying it, and evaluating its results ^41^. There are applications of the EMOI model in different sectors, such as defining public policies (Pérez G, 2020) or innovations in nutritional plans (Pérez B, 2021). This research would be one of the few applications in the field of healthcare.

In conclusion, a “Change-Management Model for Health Interventions with an Intercultural Approach” was developed, with three elements that guide organizational change in healthcare services, planning for change that incorporates interculturality in health as an important axis in the care of a predominantly indigenous population, and, for the implementation of the Plan, “Change Management Meetings” are an educational and management strategy that will obtain results if they are held frequently. It is recommended that research continue, applying the change management model developed in ethnically diverse populations, in health interventions such as dengue, and in the implementation of public policies, considering that change begins with healthcare managers, with greater leadership, and facing resistance to change without losing the motivation and empowerment of healthcare teams.
