# Supplementary material for: Action-research in the development of a change management model to strengthen health interventions with an intercultural approach, Peru
Source: Rev Peru Med Exp Salud Publica. 2025 Jun 9;42(2):203–11. doi: 10.17843/rpmesp.2025.422.14134 (PMC12377882; doi:10.17843/rpmesp.2025.422.14134)
Supplement: Translation

# Translation: Investigación-acción en la construcción del modelo de gestión de cambio para fortalecer intervenciones sanitarias con enfoque intercultural, Perú

**Authors:** Gualberto Segovia-Meza, Armando Medina-Ibañez, Marco Bartolo-Marchena, Betty Monteza-Facho

**Affiliations:** 1 Ministerio de Salud, Lima, Perú.; 2 Universidad Nacional Federico Villareal, Lima, Perú.; 3 Centro Nacional de Investigación Social e Interculturalidad en Salud, Instituto Nacional de Salud, Lima, Perú.

**Keywords:** Gestión del Cambio, Interculturalidad, Salud de la Población Indígena

## Abstract

Presentamos la experiencia en el desarrollo de un modelo de gestión del cambio para fortalecer las intervenciones sanitarias con enfoque intercultural en el primer nivel de atención. Se trata de una investigación-acción, en la que participaron equipos de salud que atienden principalmente a población indígena. La metodología incluyó talleres basados en la problematización y un análisis de tipo deductivo. El estudio se llevó a cabo en 44 establecimientos de salud de seis regiones, utilizando una guía específica para construir el modelo de cambio. Como resultado, se elaboró un modelo de gestión del cambio compuesto por tres componentes principales: Modelo orientador de la gestión del cambio. Modelo de planificación para el cambio. Reuniones de gestión del cambio, fundamentadas en la Educación Permanente en Salud. Se presenta un modelo de gestión del cambio para fortalecer la prevención y control de prioridades sanitarias con enfoque intercultural, con potencial de mejorar en la eficacia de intervenciones sanitarias.

## INTRODUCCIÓN

A nivel de las Américas, un análisis en 17 países el año 2023 sobre los desafíos del sistema de salud, estima que el 34,4% de la población tenía necesidades insatisfechas en la atención de salud, situación que afectaba más al quintil más pobre. Dentro de los factores causales se identificaron la falta de confianza en el personal de salud y que los enfoques interculturales son insuficientes y existen obstáculos de tipo lingüístico ^1^. Sin duda, la pandemia por la COVID-19 tuvo un impacto negativo para los programas de salud en los países de las Américas, en una revisión sobre el impacto de la pandemia en infecciones concurrentes, se identificó que la tuberculosis (TB) y la COVID-19 fueron dos principales causas de muerte en el mundo ^2^^,^^3^.

Antes de la pandemia, la situación del sistema de salud se caracterizaba por su poca eficiencia. La OPS/OMS el 2018 realizó una comparación de incidencias de TB en pueblos indígenas de seis países de las Américas, con población general; se observó grandes diferencias entre ellas, las del grupo étnico superaban a la población general entre 2 a 10 veces más ^4^. Esta situación era resultado del un sistema de salud con serios problemas para enfrentar prioridades sanitarias. Recién en el año 2024, en una sesión de la OPS/OMS se aprueban estrategias para fortalecer las Funciones Esenciales de Salud Pública con el propósito de acelerar la transformación de los sistemas de salud del 2024-2034 ^5^.

Un reto en el Perú es la interculturalidad en salud; somos un país pluricultural y pluriétnico, con 47 lenguas originarias, un rico patrimonio arqueológico. Según el censo del 2017, se determinó que el 24,9% de la población general se auto identificó como indígena. Esta diversidad nos obliga a promover el enfoque intercultural, con diálogos interculturales, respetuosos y de mutuos aprendizajes ^6^^,^^7^. Se tiene normatividad que promueve la interculturalidad en salud; sin embargo, hay debilidades en su aplicación.

Los pueblos indígenas son considerados población vulnerable. El Ministerio de Salud el año 2022 evaluó la gestión sanitaria del programa control de la tuberculosis en la población indígena 2017-2022, se identificó que 1,461 personas eran indígenas, un 59,3% indígenas amazónicos y un 40,7% indígenas andinos, hubo un incremento del número de casos del 2019 al 2022, relacionados a los efectos de la pandemia por la COVID-19 ^8^.

Un elemento central de los sistemas de salud eficientes es la Atención Primaria de la Salud; se necesita promover el desarrollo de nuestros sistemas de salud para mejorar la equidad, y avanzar hacia la Salud Universal (OPS/OMS). Las falencias de nuestro sistema de salud se hicieron evidentes con la presencia de emergencias sanitarias, como la viruela del mono el año 2022, o el brote de dengue del año 2023, donde nuestro sistema de salud tuvo dificultades en responder integralmente. En pocas semanas, la mayoría de las regiones del Perú reportaron casos confirmados de Viruela del mono, y en dengue llegamos a notificar 381 fallecidos ^9^^,^^10^.

Una evaluación de la gestión sanitaria en el primer nivel de atención en el Perú (2022), en establecimientos de salud que atienden a población principalmente indígena identificó barreras en la prevención y control de la pandemia por la COVID-19, estás fueron barreras en la visión, gestión y presupuesto en la atención a población indígena y afroperuana, y recomendó aplicar un modelo de gestión del cambio con enfoque intercultural, con el propósito de lograr mayor acceso a los servicios de salud ^11^. Ese mismo año 2022, la OPS/OMS hace recomendaciones para transformar los sistemas de salud y sugiere implementar el Modelo de Gestión del Cambio basado basados en los pasos de Kotter ^12^.

La gestión del cambio significar impulsar una nueva forma de desarrollo organizacional con la finalidad de mejorar su productividad o proporcionar un mayor nivel de vida, desarrollar este proceso transformador involucra muchos agentes de cambio, donde el equipo de trabajo lidera el plan a introducirse ^13^. Según Kotter y Bridges, este procedimiento transformador atraviesa pasos que requieren un tiempo considerable, un error en cualquier fase tiene efecto devastador, y los cambios incluso deben darse al interior de las personas ^14^^,^^15^. Asimismo, se vienen probando estrategias de innovación en salud, como el Modelo de Gestión de la Innovación Organizacional (EMOI), que permite entender las necesidades de los clientes, definir la innovación y aplicarla con un plan de innovación ^16^.

Actualmente, no contamos con un modelo de gestión claramente definido para promover un cambio organizacional del sector salud, el aporte de la OPS/OMS el año 2022, a partir del foro regional para ampliar el acceso equitativo a los servicios de salud, recomendó utilizar estrategias de gestión del cambio, con evidencia demostrada, adecuando los ocho pasos para liderar el cambio de Kotter ^15^. Una experiencia nacional se realizó el año 2021, el equipo de investigadores del Centro Nacional de Salud Intercultural adecuó un Modelo de Gestión del Cambio, para incorporar el enfoque intercultural en la intervención contra la tuberculosis en la región de Ica ^17^.

En base a lo descrito, el objetivo de esta comunicación es presentar la experiencia en el desarrollo de un modelo de gestión del cambio para fortalecer intervenciones sanitarias con enfoque intercultural.

## ENFOQUE METODOLÓGICO

El presente artículo describe el proceso de desarrollo de un modelo de gestión del cambio, utilizando la investigación-acción, que tiene como base epistemológica al constructivismo, permitiendo comprender la construcción del conocimiento por medio de la práctica, con el propósito de fortalecer las intervenciones sanitarias con un enfoque intercultural ^18^^-^^21^.

### Población y lugar de estudio

El estudio se desarrolló en regiones seleccionadas con mayor concentración de poblaciones indígenas. La población de estudio estuvo constituida por equipos de salud de establecimientos del primer nivel de atención, que fueron convocados a talleres programados para analizar y proponer planes de cambio para intervenciones sanitarias priorizados por los mismos equipos de salud.

La selección de establecimientos de salud participantes se realizó por un muestreo de tipo intencional, con criterios de ser establecimientos de salud que atienden población mayoritariamente indígena andina, amazónica. El método de abordaje fue una convocatoria oficial del Centro Nacional de Salud Intercultural a las Direcciones Regionales de Salud (DIRESA), y estas convocan y priorizan a establecimientos de salud que atiendes a población indígena principalmente. El tamaño de la muestra fue de 44 establecimientos de salud del primer nivel de atención, de 6 regiones intervenidas: Ica (2021-2022), Centro de Salud La Tinguiña, Parcona, Guadalupe, Puesto de Salud Pampa Villacuri; Redes de Salud: Chincha, Pisco, Ica, Nasca. En Amazonas (2022), las Redes: Chachapoyas, Bagua y Condorcanqui, Utcubamba, IPRESS que atienden población Awajun y Wampis. Ayacucho (2022) en Microred Vinchos, Microred Morochucos, Microred Ocros, atienden población quechua principalmente. Pasco (2022), con Red Oxapampa. Junín (2022) a Instituciones Prestadoras de Servicios de Salud (IPRESS) de Pangoa, Pichanaki, Satipo. Huánuco (2022) a las IPRESS de Leoncio Prado, Puerto Inca, que atienden comunidades Ashaninkas, Yaneshas.

### Recolección de datos

Las intervenciones o estrategias sanitarias fueron definidas por cada establecimiento de salud, en otros, lo definió la DIRESA de acuerdo con su prioridad regional. Para el desarrollo de los talleres se utilizó la metodología de la problematización, que inicia con la observación de la realidad del problema, luego se identifica los puntos críticos, la teorización del tema, se plantean hipótesis de solución. El programa de los talleres tuvo una duración de 2 días, y permitió a los investigadores obtener información con la técnica observación-participante, que consistió en la participación de los investigadores en las discusiones de grupos, con preguntas motivadoras para propiciar la problematización de temas.

Los datos fueron recolectados de los papelógrafos generados por los equipos de salud, de las plenarias realizadas, grabaciones, registro de las guías de planes de cambio, entrevistas a jefes de establecimientos de salud, informes de los talleres, observaciones al desarrollo de los planes, de las presentaciones virtuales realizadas.

Toda la información recolectada fue sistematizada con base a los objetivos del estudio, o sea, en los componentes del modelo de gestión del cambio en desarrollo: a) Modelo orientador de la gestión del cambio. b) Modelo de planificación para el cambio. c) Reuniones de gestión del cambio basado en la Educación Permanente en Salud (EPS).

### Procedimiento

Un primer momento. Se inició con un plan para la región Ica (2021), “Desarrollo de un Modelo de Gestión del Cambio para fortalecer la prevención y control de la tuberculosis con enfoque intercultural”. Con la participación de la autoridad sanitaria regional se desarrollaron talleres programados con los equipos de salud de 4 establecimientos de salud; se utilizó la metodología de investigación-acción, considerando los siguientes pasos: 1. Un primer ciclo: Donde se analizó la situación actual de la gestión integral del programa de tuberculosis, utilizando la pedagogía de la problematización, y los equipos de salud identifican sus problemas y necesidades respecto a las necesidades de los usuarios, las necesidades de la organización, gestión de los recursos, y la prestación de servicios e interculturalidad. 2. Segundo ciclo. Se desarrolló un plan de fortalecimiento en la prevención y control de la tuberculosis con enfoque intercultural, utilizando una guía adecuada del modelo de gestión de la innovación organizacional (EMOI). 3. Tercer ciclo. Se capacitó a los equipos de salud y jefes de establecimiento en ejecutar el plan; se utilizó una adecuación de la estrategia de la Educación Permanente en Salud (EPS) para ejecutar el plan elaborado. 4. Cuarto ciclo. Se evaluó las acciones de implantación y efectos, se realizaron mejoras o ajustes al diseño original del Modelo en desarrollo.

Segundo momento. Se desarrolló un plan de acción para implementar el desarrollo del “Modelo de Gestión del Cambio para Intervenciones Sanitarias con enfoque intercultural”, en las regiones: Ayacucho, Amazonas, Pasco, Huánuco, Junín. Se desarrollaron las actividades según el diseño de la investigación-acción; se siguió un primer ciclo, con detectar los problemas en la organización, gestión y prestación de las intervenciones sanitarias priorizadas por los equipos de salud. Los temas abordados fueron: COVID-19, TB, VIH/SIDA, dengue, salud materna, salud mental, viruela del mono; luego se desarrollaron el segundo, tercer y cuarto ciclo, recolectando los datos en cada uno de ellos, su análisis y sistematización, orientados al desarrollo del modelo de estudio.

Tercer momento. Se definió el “Modelo de Gestión del Cambio para Intervenciones Sanitarias con enfoque intercultural”, con toda la información recopilada se realiza la codificación de datos cualitativos en torno al problema de investigación. ¿Cuáles son los elementos del modelo de gestión del cambio como herramienta que permitirá fortalecer las intervenciones sanitarias con enfoque intercultural? Se realizó la categorización para su análisis a través del análisis deductivo, en forma manual, e interpretación de los datos, de acuerdo con los fenómenos del estudio, definidos como categorías, que son tres: 1. Análisis de involucrados y de la gestión 2. Planificación para el cambio. 3. Definiendo el modelo de gestión del cambio (Figura 1).

**Figura 1:** Árbol de codificación para el estudio: Tema, categorías y subcategorías.

### Análisis de datos

Número de codificadores. Los datos recopilados fueron codificados por el investigador principal, y la asistencia de 3 investigadores del CENSI. Se realizó una codificación manual y a través del programa Excel.

Descripción del árbol de codificación. Se inició identificando los temas de investigación, luego se han creado las categorías principales, finalmente las subcategorías que se muestran en la Figura 1.

Comprobación por los participantes. Los participantes, durante el desarrollo de los talleres regionales expusieron el análisis del problema relacionado a los pasos del modelo de gestión del cambio adecuado para el presente estudio. Luego proporcionaron información sobre los hallazgos de su análisis en las necesidades de los usuarios, de la gestión de sus recursos, y las dificultades en la elaboración de los planes de cambio. Durante las asistencias técnicas presencial y virtual los equipos de salud, mostraron planes de cambio culminados, y en algunos comprobamos su implementación.

### Reportes

Modelo de gestión del cambio adecuado para la intervención. Se le denomina “Modelo de gestión del cambio para intervenciones sanitarias con enfoque intercultural” y tiene tres componentes:

1. Modelo orientador de la gestión del cambio. Luego de los ajustes correspondientes con base en la retroalimentación de los equipos de salud, se consolida como componente orientador en todo el proceso de desarrollo de la gestión del cambio, se adecuó del modelo de Kotter, y del modelo propuesto por OPS/OMS ^1^^,^^14^ (Figura 2).

**Figura 2:** Adecuación del Modelo de Gestión del Cambio para la intervención como “Modelo orientador de la Gestión del Cambio”

2. Modelo de planificación para el cambio. Se adecuó a partir del EMOI, este modelo de gestión tiene 4 componentes: El componente 1 (C1), es de análisis de involucrados y equipo de gestión, análisis de las necesidades de los usuarios, y de la organización, gestión y prestación de los servicios. Componente 2 (C2), se desarrolla el plan con sus componentes de organización, gestión, incorpora los 4 ejes claves: gestión de recursos humanos en salud, Sistemas de información, Gestión clínica e Interculturalidad, en especial la acción sobre registro correcto de los códigos étnicos. Componente 3 (C3), representa el espacio para el desarrollo del plan y sus ejes en recursos humanos, sistema de información, gestión clínica e interculturalidad. Componente 4 (C4), se evalúan los cambios generados (Figura 3).

**Figura 3:** Modelo de Gestión del Cambio para Intervenciones Sanitarias con enfoque intercultural.

3) Reuniones de gestión del cambio. Se diseño tomando como base la Reflexión Crítica de la Práctica de la Educación Permanente en Salud (EPS), que es una estrategia educativa y de gestión. Estas reuniones representan el motor del cambio, los equipos de salud acuerdan las fechas, y ambientes de sus reuniones de gestión, se ha considerado un espacio para dialogar sobre la ejecución del Plan, en ese espacio se comparte experiencia y se produce la educación permanente en salud, y los cambios planteados.

### Citas de participantes

En el proceso de construcción del Modelo de Gestión del Cambio para Intervenciones Sanitarias con enfoque intercultural, se realizaron talleres de problematización, recopilación de datos, y sistematización de información, de acuerdo con las categorías y subcategorías establecidas (Tabla 1).

**Tabla 1:** Modelo resumido por categorías, subcategorías, hallazgos y lecciones aprendidas.

Categoría	Subcategoría / Preguntas para el análisis.	Hallazgos (Citas)	Lecciones aprendidas (Resumen de investigadores)
1. Análisis de involucrados y de la gestión	1.1 Identificando a los impulsores del cambio y equipo de cambio.		
	¿Cómo se establece la sensación de urgencia al cambo en los gestores y equipo técnico en las DIRESAS/REDES DE SALUD?	*Las autoridades sanitarias regionales y provinciales tienen un desconocimiento de la gestión del cambio. No se valora como importante la interculturalidad en los servicios de salud, nos falta más capacitación.* (Jefa CS Guadalupe, Ica)	En la DIRESA y las Redes de Salud la motivación inicial para el cambio se reduce si no hay acciones que fortalecen el liderazgo, trabajo en equipo y mejoras en la comunicación. Hay más interés en los equipos de gestión de las IPRESS sobre la urgencia en implementar cambios.
	¿Cómo está conformado el equipo conductor del programa de prevención y control de la TB y dengue en las IPRESS del primer nivel de atención?	*“Equipo de gestión para el control de la TB, lo conforman los responsables de la estrategia de TB y los contratados”.* (jefe PS Pampa Villacuri, Ica) *“En dengue, el equipo es el responsable de metaxénicas, promoción de la salud, y salud ambiental principalmente”.* (CS Chincha Baja, Ica)	Los equipos de gestión de las estrategias sanitarias en su mayoría no incorporan a otros actores claves en el proceso de atención de pacientes, como responsables de farmacia, laboratorio, consultorios externos, incluidos los ACS, y líderes de la comunidad.
	1.2 Análisis de las necesidades de los usuarios		
	¿Realizan la identificación de necesidades de los usuarios con TB, dengue, COVID-19 para mejorar su salud? ¿Con enfoque de etnicidad? (utilizando el mapa de empatía).	*“Los EE.SS. no aplican estrategias para identificar la necesidad de los usuarios con TB, dengue, COVID-19.”* (funcionario de Red de Salud de Ayacucho) *Al aplicar el mapa de empatía permite identificar problemas en el acceso a los servicios de salud, si no se tiene una encuesta al usuario”.* (Ponente del PS, Amazonas)	El mapa de empatía permite conocer mejor las necesidades de los pacientes con TB, COVID-19, dengue, VIH/SIDA y permite identificar nuestras debilidades en responder mejor a las necesidades con mayor pertinencia étnica.
	¿Cuáles son las necesidades de los familiares de pacientes con TB, con enfoque de etnicidad? (utilizamos el mapa de empatía).	*“No se cuenta con información sobre los conocimientos y percepciones de la familia del paciente con TB, y su identidad étnica”.* (Lic. del PCT del Centro de Salud Parcona, Ica)	Se reconoce que la familia tiene temores de contagio, baja la economía familiar, ideas de retornar a su lugar de origen “su pueblo”. Personal de salud evidencia que no tiene competencias en intervención familiar, y tampoco del enfoque intercultural.
	¿Cuáles son las necesidades de los usuarios internos (personal de salud), para una mejor calidad en la atención de los usuarios de las Estrategias Sanitarias?	*“Identificamos las limitaciones u obstáculos que tiene el personal o equipo de salud de la estrategia sanitaria para cumplir la normatividad correspondiente. Así como la relación con las unidades y servicios de esta IPRESS, o dificultades para el trabajo con las familias y la comunidad”.* (Exposición del Equipo de salud del Red de Salud Bagua, Amazonas).	La metodología permite reflexionar sobre debilidades en el trabajo multidisciplinario, valorar la interculturalidad en salud para mejorar el acceso a los servicios de salud. Reconocen debilidades en interculturalidad y en especial en el registro correcto de los códigos étnicos.
	1.3 Organización y gestión de los servicios de salud		
	¿Cómo se organiza y gestiona las IPRESS para vencer obstáculos, delegar responsabilidades y producir los cambios al enfrentar a la TB, y dengue?	*Los equipos de trabajo de TB, dengue, realizan su trabajo generalmente en forma aislada, o fragmentada, El equipo son los contratados para TB”. “El equipo para el control del dengue son los coordinadores de metaxénicas, salud ambiental y promoción de la salud”.* (CS La Tinguiña, Ica)	Organizar equipos de trabajo con los involucrados en el proceso de brindar los cuidados integrales en casos de TB, dengue, Se necesita decisión y liderazgo de los jefes de IPRESS y gestores de Redes y DIRESA, para darle sostenibilidad a los equipos.
	¿Cuál es la situación de su sistema de información para las estrategias sanitarias de TB, dengue, así como el registro de los códigos étnicos, para las emergencias sanitarias como la COVID-19, dengue y TB?	*“Las IPRESS no cuentan con historia clínica electrónica. La estrategia sanitaria de TB tiene un programa SIGTB que no cuenta con los códigos étnicos correctos. Las IPRESS tienen para el dengue el mapa de calor, pero se utiliza muy poco para la intervención. No se cuentan con salas situacionales desagregados por grupos étnicos”.* (Exposición del equipo del PS Pampa Villacuri-Ica)	Las capacitaciones en los registros correctos de los códigos étnicos han identificado a personas con TB de los pueblos indígenas (en Ica: Puesto de Salud Pampa Villacuri: Shipibos y Quechuas), y se ha promovido acciones para mayor pertinencia cultural. Poco uso de los mapas de calor en dengue. No uso de datos de distritos con baja cobertura de vacuna contra la COVID-19.
2. Planificación para el cambio	2.1 El Plan de cambio para la intervención sanitaria		
	¿Es posible contar un Plan de Cambio para fortalecer la prevención y control de la prioridad sanitaria, y ejecutarlos?	*“Ahora tenemos un Plan de cambio más participativo, pero el lento desarrollo del Plan y no ejecución de tareas, está relacionado a pocas reuniones de gestión. Los equipos técnicos están más comprometidos e interesados en implementar mejoras como registro de códigos étnicos, siempre hay los que se oponen a los cambios que se quieren implementar”.* (Exposición del equipo del PS Pampa Cangallo-Ayacucho).	Gestores de DIRESA, y Redes de Salud no se empoderan, se necesita un plan específico para funcionarios y gestores en liderazgo, gobernanza y cambio organizacional. La sostenibilidad de lo avanzado por los equipos técnicos requiere acompañamiento y recursos para ejecutar el Plan de Cambio.
	¿Cuáles son los puntos críticos en el desarrollo del Plan de cambio, y las reuniones de gestión del cambio para la prevención y control de prioridades sanitarias?	*“No se realizan reuniones de gestión en forma regular en las IPRESS, para abordar los problemas en la prevención y control de las estrategias sanitarias porque los horarios no coinciden de los equipos de salud, se está pensando en otras estrategias para programar mejor los horarios y otros”.* (Jefa CS Sunampe, Ica).	Las reuniones se hacen efectivas cuando hay convocatoria formal, inicia siempre con una reflexión crítica de la práctica. Necesario realizar capacitaciones en la estrategia de reuniones de problematización o de educación permanente en salud.
3. Definiendo el Modelo de Gestión del Cambio	3.1 Desarrollo del Modelo con enfoque intercultural		
	Estableciendo la visión de cambio organizacional.	*“Al 2025 las IPRESS de Nieva, Urakusa y Pagkintsa de la MR Nieva a través de sus equipos impulsores del cambio lograrán promover la captación, prevención y atención con oportunidad y calidad los casos de VIH-SIDA con la participación y vigilancia de la comunidad con un enfoque intercultural”* (Equipo de salud Centro de Salud Nieva, Centro de Salud Urakusa y Centro de Salud Pagkintsa. Amazonas).	El instrumento (Guía) para el desarrollo del Plan de Cambio con los componentes del modelo, permite el desarrollo del Plan de Cambio sobre las prioridades sanitarias locales, con una visión cambio organizacional y enfoque intercultural.

## DISCUSIÓN

Los resultados muestran el desarrollo de un Modelo de Gestión del Cambio para Intervenciones Sanitarias con enfoque intercultural, que contiene tres elementos. En su desarrollo se siguió el esquema de una investigación-acción, con un primer momento de análisis del problema. Segundo momento: elaborar el plan. Tercer momento: implementar y evaluar el plan. Cuarto Momento: de retroalimentación, en la cual se revisó y se hizo los ajustes necesarios al modelo.

Desde inicios del siglo XXI se recomendaba sobre la gestión de un cambio organizacional en el sector público, en cómo integrar el conocimiento obtenido a partir de múltiples perspectivas teóricas a la práctica, y desarrollar mejores modelos y herramientas para la investigación del cambio ^19^. Una tarea nada fácil, nosotros revisamos la metodología de investigación-acción en el campo de la salud, se cuenta con algunas publicaciones en salud, pero sin duda, existen más aplicaciones en el campo de las ciencias sociales ^22^^-^^25^.

Esta metodología de la investigación-acción tiene como propósito aportar información para propiciar el cambio social, o transformar la realidad. O sea, permite vincular el estudio de los problemas sociales, y se logren de forma simultánea conocimientos y cambios sociales ^20^. Nuestro estudio, utiliza la metodología de investigación-acción para concentrar su atención en comprender y explicar el proceso de desarrollo de un modelo de gestión del cambio para fortalecer las intervenciones sanitarias con enfoque intercultural, que debería conducir a una mejora de eficacia en la prevención y control de los problemas sanitarios, en especial en poblaciones indígenas.

Un tema central de la investigación es el desarrollo estudios sobre Modelos de Gestión del Cambio en salud, al año 2022 se realizó una revisión bibliográfica sobre el tema “gestión del cambio en el marco de la gestión de la seguridad y salud en el trabajo”, se evidenció, en general, escasos estudios sobre la temática, y se recomendó mayores estudios ^21^. Otros estudios trataron sobre las Tecnologías Móviles para Gestión del Cambio en organizaciones sanitarias ^22^, existe el curso virtual que OPS/OMS desarrolla “Gestión del Cambio para servicios de Telesalud” (2024), y que define al modelo de gestión del cambio como “la aplicación de un proceso estructurado y un conjunto de herramientas para liderar el lado humano del cambio y alcanzar los resultados deseados”. En tal sentido, hay poca información sobre modelos de gestión del cambio adecuados para fortalecer las intervenciones sanitarias con enfoque intercultural ^26^^-^^29^.

Se hace necesario clarificar la importancia de un modelo de gestión del cambio para potenciar el impacto de intervenciones sanitarias. Los trabajos de Kotter en el campo de organizaciones privadas con la experiencia en renovar empresas, señala que los “líderes que transforman con éxito a sus empresas hacen correctamente ocho pasos de la gestión del cambio, y los hacen en el orden correcto” ^14^. No se encontró aplicaciones de Kotter en el campo sanitario, un acercamiento más concreto es el documento “Gestión del Cambio en la Salud Pública” de OPS/OMS (2022), que define a la gestión del cambio como, la práctica y el proceso de apoyar a las personas durante un cambio, y se enfatiza en ayudar a las personas en modificar sus comportamientos, actitudes o procesos del trabajo para lograr el objetivo deseado ^2^^,^^30^^,^^31^. Luego en el documento Ampliación del Acceso equitativo a los Servicios de Salud: Recomendaciones para la transformación de los sistemas de salud hacia la salud universal; propone una adecuación de los ocho pasos de Kotter ^1^. (Figura 2), y recomienda su aplicación en el campo sanitario.

En tal sentido, planteamos el desarrollo de un modelo de gestión del cambio, tomando como base los ocho pasos de Kotter, y las adecuaciones realizadas por la OPS/OMS. Agregamos dos elementos complementarios, una adecuación al EMOI ^32^^-^^38^ para desarrollar un plan de cambio; y una estrategia para operativizar el plan “Las Reuniones de Gestión del Cambio”, que representa una estrategia educativa y de gestión para los servicios de salud, con una metodología basada en la Educación Permanente en Salud, desarrollada por María C. Davini (OPS) como reuniones de Reflexión Crítica de la Práctica ^23^.

Sobre la interculturalidad en salud, las revisiones señalan la existencia de normatividad aprobada sobre el tema, desde el sector salud y cultura principalmente, el problema se concentra en ponerse en práctica ^39^. Uno de sus problemas es su complejidad de articulación con la medicina científica, hay problemas en entender los conceptos como el síndrome cultural que no es fácilmente comprendida por los operadores de la medicina convencional ^40^. Durante el desarrollo se identificó algunos puntos claves de no aplicar la normatividad ^25^, como la autoidentificación étnica, y registro correcto de los códigos étnicos, seguido del no análisis de la información y uso de información con la variable étnica para implementar mejoras. Una experiencia interesante representa el Puesto de Salud Pampa Villacuri en Ica, el equipo de salud se empoderó y comprendió la necesidad de conocer las necesidades de población migrante. En los últimos años, realizaron un registro correcto de los códigos étnicos, análisis de datos, una sala situacional con enfoque intercultural se identificó que la población quechua y shipiba eran los principales afectados de tuberculosis ^17^. Por lo tanto, en nuestro modelo de gestión del cambio ubicamos a la interculturalidad como un objetivo clave al interior del plan de cambio.

Respecto a la relación del primer componente del modelo, “Modelo orientador de la gestión del cambio”, con el segundo componente, “Modelo de planificación para el cambio”, el primer modelo contiene los 8 pasos adecuados del trabajo de Kotter y permite direccionar el proceso de cambio organizacional. En su tercer paso, “Desarrollo de un Plan con una visión estratégica” (Figura 3), se incluye el desarrollo del plan de gestión del cambio que ayuda a gestionar el proceso de cambio y articular claramente la estrategia organizativa ^41^^,^^42^. Esta articulación de dos modelos ha permitido desarrollar el plan de cambio de los establecimientos de salud, que considera la programación de actividades de los pasos iniciales principalmente, y en el tercer paso se incorpora el desarrollo de todos los componentes del “Modelo de Planificación para el Cambio”. Según el Centro de Gestión de Calidad y del Cambio de la Universidad Politécnica de Valencia, define al modelo EMOI como una metodología que permite el despliegue efectivo de la innovación tanto a nivel estratégico como táctico u operativo, y que permite entender las demandas de innovación, definir la innovación, desplegarla y evaluar sus resultados ^41^. Existe aplicaciones del modelo EMOI en diferentes sectores como definir políticas públicas (Pérez G, 2020), o en innovaciones en planes nutricionales (Pérez B, 2021), esta investigación sería una de las pocas aplicaciones en el campo de la salud.

En conclusión, se desarrollo un “Modelo de Gestión del Cambio para intervenciones sanitarias con enfoque intercultural”, con tres elementos, que permiten orientar un cambio organizacional de servicios de salud, una planificación para el cambio, que incorpora a la interculturalidad en salud como un eje importante en la atención a población mayoritariamente indígena, y para la ejecución del Plan, las “Reuniones de Gestión del Cambio” son una estrategia educativa y de gestión que permitirá el logro de resultados si se desarrollan frecuentemente. Se recomienda continuar investigaciones aplicando el modelo de gestión del cambio desarrollado; en poblaciones étnicamente diversas, en intervenciones sanitarias como el dengue, e implementación de políticas públicas, considerando que el cambio inicia en los gestores de salud, con mayor liderazgo, y enfrentando la resistencia al cambio sin perder la motivación y empoderamiento de los equipos de salud.
